# Metabolic flux analysis of secondary metabolism in plants

**DOI:** 10.1016/j.mec.2020.e00123

**Published:** 2020-02-01

**Authors:** Meng-Ling Shih, John A. Morgan

**Affiliations:** Davidson School of Chemical Engineering, Purdue University, West Lafayette, IN, 47907, USA

**Keywords:** Metabolic flux analysis, Plant secondary metabolites, Subcellular compartmentation, Stable isotopic labeling, Metabolic channeling, BA, Benzoic acid, DMAPP, Dimethylallyl diphosphate, GC, Gas chromatography, LC, Liquid chromatography, IP, Isopentenyl phosphate, IPP, Isopentenyl diphosphate, MFA, Metabolic flux analysis, INST-MFA, Isotopically non-steady state metabolic flux analysis, MVA, Mevalonic acid, MVAP, Mevalonate 5-phosphate, MVAPP, Mevalonate 5-diphosphate, MEP, Methylerythritol 4-phosphate, MS, Mass spectrometry, NMR, Nuclear magnetic resonance, Phe, Phenylalanine, ^13^C MFA, Steady state isotopically labeled metabolic flux analysis

## Abstract

Numerous secondary metabolites from plants are important for their medicinal, nutraceutical or sensory properties. Recently, significant progress has been made in the identification of the genes and enzymes of plant secondary metabolic pathways. Hence, there is interest in using synthetic biology to enhance the production of targeted valuable metabolites in plants. In this article, we examine the contribution that metabolic flux analysis will have on informing the rational selection of metabolic engineering targets as well as analysis of carbon and energy efficiency. Compared to microbes, plants have more complex tissue, cellular and subcellular organization, making precise metabolite concentration measurements more challenging. We review different techniques involved in quantifying flux and provide examples illustrating the application of the techniques. For linear and branched pathways that lead to end products with low turnover, flux quantification is straightforward and doesn’t require isotopic labeling. However, for metabolites synthesized via parallel pathways, there is a requirement for isotopic labeling experiments. If the fed isotopically labeled carbons don’t scramble, one needs to apply transient label balancing methods. In the transient case, it is also necessary to measure metabolite concentrations. While flux analysis is not able to directly identify mechanisms of regulation, it is a powerful tool to examine flux distribution at key metabolic nodes in intermediary metabolism, detect flux to wasteful side pathways, and show how parallel pathways handle flux in wild-type and engineered plants under a variety of physiological conditions.

## Highlights

• Plant secondary metabolites have high economic value to human health and pleasure.
• Plant secondary metabolites are synthesized by pathways in subcellular compartments.
• Metabolic flux analysis can guide the selection of metabolic engineering targets.

## 1 Introduction

**Fig. 1:**
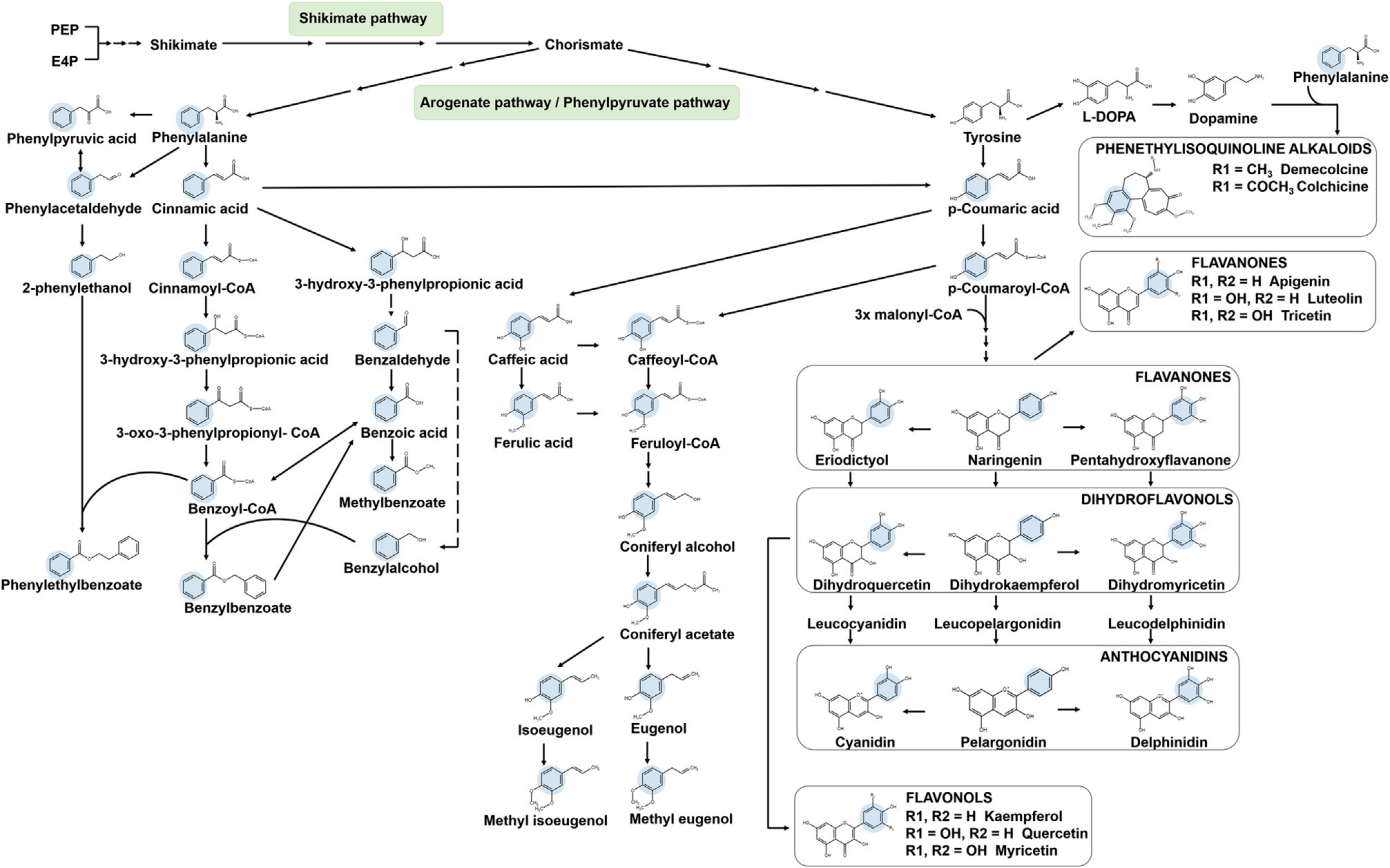
Plant phenylpropanoid/benzenoid, polyketide, and phenethylisoquinoline alkaloid secondary metabolic pathways. The shaded areas represent the portion of the carbon backbone that does not undergo rearrangement.

Plant secondary metabolites are defined as the natural products found in selected plants that are beneficial for their survival and reproduction in the environment. Secondary metabolites are derived from building blocks synthesized in primary and intermediary metabolism, such as aromatic amino acids from the shikimate pathway (Fig. 1), or isopentenyl diphosphate (IPP) and dimethylallyl diphosphate (DMAPP) from the isoprenoid pathway. As a kingdom, plants are capable of producing over 200,000 distinct natural products for specialized functions related to defense (Hiltpold and Turlings, 2012; Huang et al., 2012; Johann et al., 2011; Kappers et al., 2011) and reproduction (Luft et al., 2003; Raguso, 2008). Several of these chemicals have high value to humans as pharmaceuticals (Benowitz, 2009; Montanher et al., 2007; Yi et al., 2004; Zbigniew et al., 2014), nutraceuticals (Naveen and Baskaran, 2018), colorants, flavors and fragrances, thus making them targets for metabolic engineering.

Although there have been impressive advances in the ability to engineer plant pathways into microbial hosts for secondary metabolite production (Dziggel et al., 2017; Jiang et al., 2005; Leonard et al., 2007; Li et al., 2018; Minami et al., 2008; Nakagawa et al., 2011; Pyne et al., 2019; Westfall et al., 2012), due to economic considerations, the current source of most of these metabolites is from plants. Therefore, an accurate understanding of the plant physiology is important for understanding how carbon, nitrogen and chemical energy are distributed to the biosynthesis of secondary metabolites. Comparing the actual carbon and energy used to the theoretical requirements, respectively, are key measurements of pathway efficiency. Metabolic flux analysis (MFA) is a method to quantitatively estimate the intracellular flows of carbon throughout the myriad of metabolic networks. MFA produces detailed flux maps which are an integrated measure of the cellular phenotype as they are the result of transcriptional, translational, and allosteric regulatory mechanisms (Stephanopoulos et al., 1998).

### 1.1 Rationale for quantifying fluxes in plant secondary metabolism

**Fig. 2:**
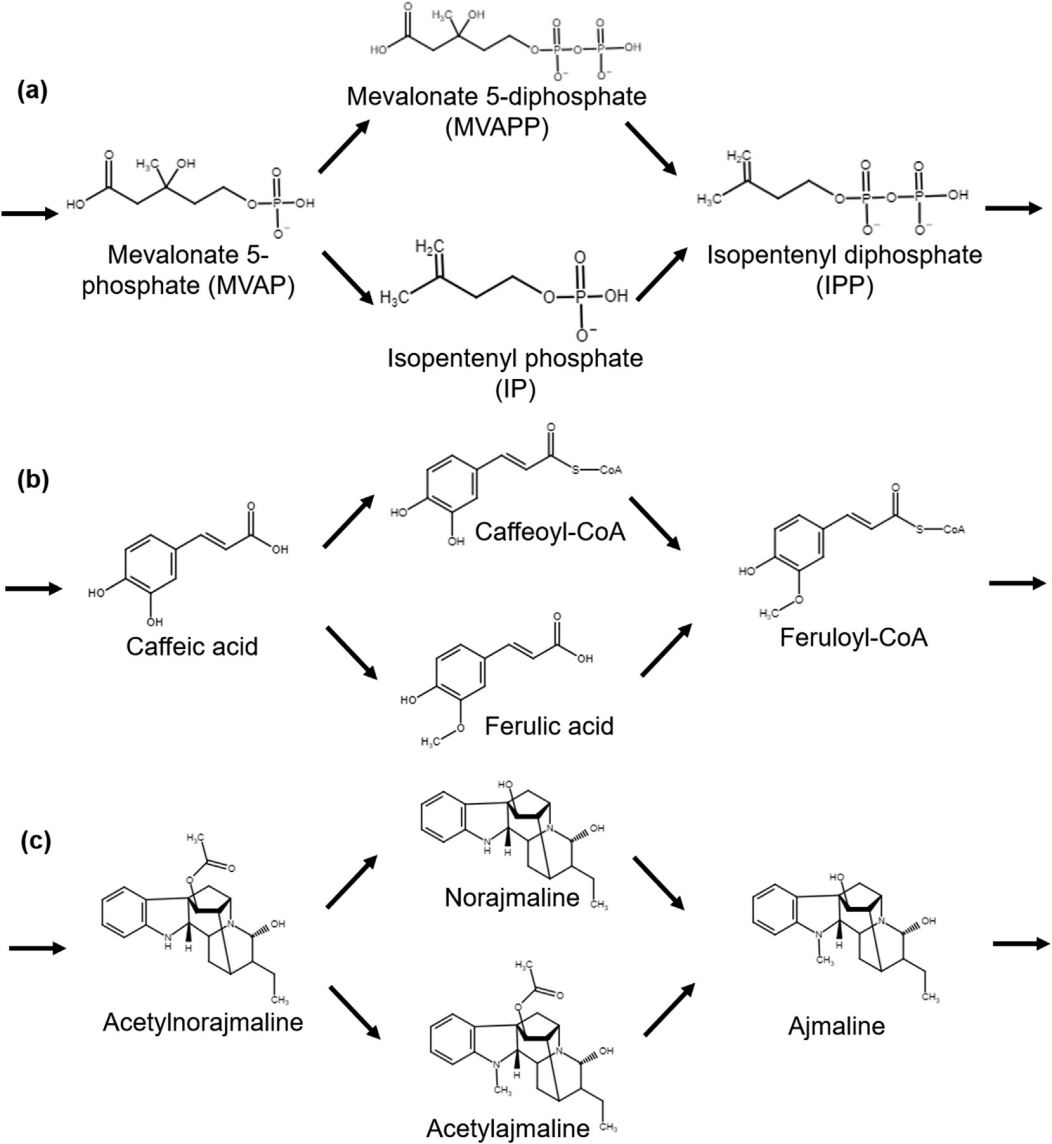
Examples of the pathway split which will converge to the same product downstream. (a) The formation of isopentenyl diphosphate (IPP) from mevalonate 5-phosphate (MVAP) through mevalonate 5-diphosphate (MVAPP) or isopentenyl phosphate (IP) in the mevalonic acid (MVA) pathway (Henry et al., 2015). (b) The formation of feruloyl-CoA from caffeic acid through caffeoyl-CoA or ferulic acid in the phenylpropanoid pathway (Bonawitz and Chapple, 2010). (c) The formation of ajmaline from acetylnorajmaline through norajmaline or acetylajmaline in the monoterpenoid indole alkaloids biosynthetic pathway (Ziegler and Facchini, 2008).

In plant secondary metabolism, evolution has led to parallel pathways that can synthesize a particular metabolic intermediate. Some examples of this type of network are shown in Fig. 2. Resolving the flux contributions of each pathway can be difficult. Methods used to address this issue include applying enzyme specific inhibitors, gene knockdown, or *in vitro* enzyme kinetic characterization. The results of these experiments are used to make estimates of the relative contribution of enzymes competing for the same substrate, and thus the flux split at a given branch point. However, the use of inhibitors or gene knockdown experiments may have off-target effects and perturb the cellular system. In contrast, MFA is an approach to quantify the flux splits under the physiological condition.

Another major reason for performing MFA in plants is the quantification around key intermediary precursors of secondary metabolites. For example, quantifying the flux around acetyl-CoA is important for determining the amount going towards fatty acids vs. terpenoids. Another example is solving mass balances around phenylalanine to compare the flux to protein synthesis vs. phenylpropanoid metabolism.

Additionally, within secondary metabolism it is imperative to identify and quantify the main competitive branches that lead flux away from the secondary metabolite of interest. In this manner, the ‘wasteful fluxes’ leading to undesired side products can be identified and subjected to metabolic engineering for elimination. Therefore, MFA is a powerful method for investigating plant secondary metabolic networks in a systematic and quantitative manner.

### 1.2 Plants have complex localization of metabolic pathways

Eukaryotes have a more complex spatial distribution of metabolic pathways than bacteria (Wahrheit et al., 2011), which poses a major challenge for flux analysis. Plants have a high degree of compartmentation at the tissue and subcellular levels with metabolite pools and enzymes in multiple locations and parallel pathways in different organelles, which increases metabolic flexibility (Allen et al., 2009). This complexity affects two measurements commonly used in MFA (see section 2.2). First, metabolite concentrations are quantified as the total moles divided by the cellular volume. For metabolites that occur only in specific subcellular location(s), this analysis would lead to an underestimation of the true concentration. Second, metabolite labeling patterns can be different for the same metabolite in different compartments. Hence this measurement is in error as it is the combination of the isotopic labeling of metabolites from each compartment. Nevertheless, if the measurements can be deconvoluted, MFA has the power to determine the relative flux distribution between the compartments.

The complexity of compartmentation can be resolved by utilizing methods to separate organelles or techniques that provide information on compartment-specific metabolites or proteins (Allen et al., 2009; Fernie and Morgan, 2013; Shachar-Hill, 2013). Resolving the fluxes of parallel metabolic routes that involve exchange of intermediates between compartments is difficult. For example, cytosolic MVA pathway and plastidic methylerythritol 4-phosphate (MEP) pathway are responsible for the biosynthesis of IPP/DMAPP which are the precursors of diverse isoprenoids and are known to be exchanged between the compartments. To measure the relative contribution of the pathways to specific isoprenoids, researchers fed various labeled precursors, either glucose, the precursor of pyruvate, acetyl-CoA and glyceraldehyde 3-phosphate (Rohmer, 1999; Schuhr et al., 2003), or the pathway-specific precursors such as mevalonate and 1-deoxy-D-xylulose (Opitz et al., 2014). From the analysis of the isoprenoid labeling patterns, the researchers were able to calculate the relative inputs from the two pathways. However, this exchange rate can be altered by the external feeding of precursors to the pathways. As argued in Opitz et al. (2014), with exogeneous supplied [2–^13^C]-mevalonolactone or [5,5-^2^H2]-1-deoxy-D-xylulose, the activity of each pathway and the magnitude of crosstalk between the pathways could vary in different organs and during different developmental stages. This may be one reason of the considerably varied labeling of isoprenoids in different experiments (Arigoni et al., 1997; Bartram et al., 2006; Dudareva et al., 2005; Fukusaki et al., 2004; Hampel et al., 2006, 2005; Kasahara et al., 2002; Schwender et al., 1997).

## 2 Metabolic flux analysis techniques

Metabolic flux is defined as the amount of a metabolite processed by one or more catalytic steps per unit of time, and it is normalized by cellular abundance (e.g. gram dry weight) (Stephanopoulos et al., 1998). Though MFA applied to several plant systems have been mostly focused on central carbon metabolism such as photosynthesis (Ma et al., 2014), cell wall formation (Chen et al., 2013) and lipid biosynthesis (Borisjuk et al., 2013) (for reviews see (Allen et al., 2009; Fernie and Morgan, 2013; Shachar-Hill, 2013)), the techniques developed for primary metabolism are powerful and can be extended to secondary metabolism. However, despite the tremendous chemical diversity, secondary metabolites represent a small fraction of the total mass of plant tissues. This means that, as a whole, the time averaged total carbon flux to secondary metabolites is typically just a few percent of the whole metabolic network of the plants, rendering it difficult to precisely measure. The theory behind flux analysis is based upon mass balances on all the inputs and outputs that are taken up and excreted by cells, respectively, and includes the growth of cells as an output. Therefore, a logical approach to estimate flux to secondary metabolism would be to measure all the outputs to cell growth (primary metabolism) and subtract the nutrient inputs. This difference would be the flux to secondary metabolism. However, the errors associated with the measurements of primary metabolic fluxes, ca. 10–15%, are often larger than the flux to all secondary metabolites. Thus, this method is not appropriate for secondary metabolism. Moreover, the production of secondary metabolites is often not a continuous process but rather induced by development or certain phenomena such as pathogen attack. Because the fluxes are much lower than primary metabolism and distributed over various tissues/organs and various developmental stages, there is a need to adapt flux analysis techniques that were developed for primary metabolism to secondary metabolism.

### 2.1 Quantification of fluxes in secondary metabolism by mass balances of end products

**Fig. 3:**
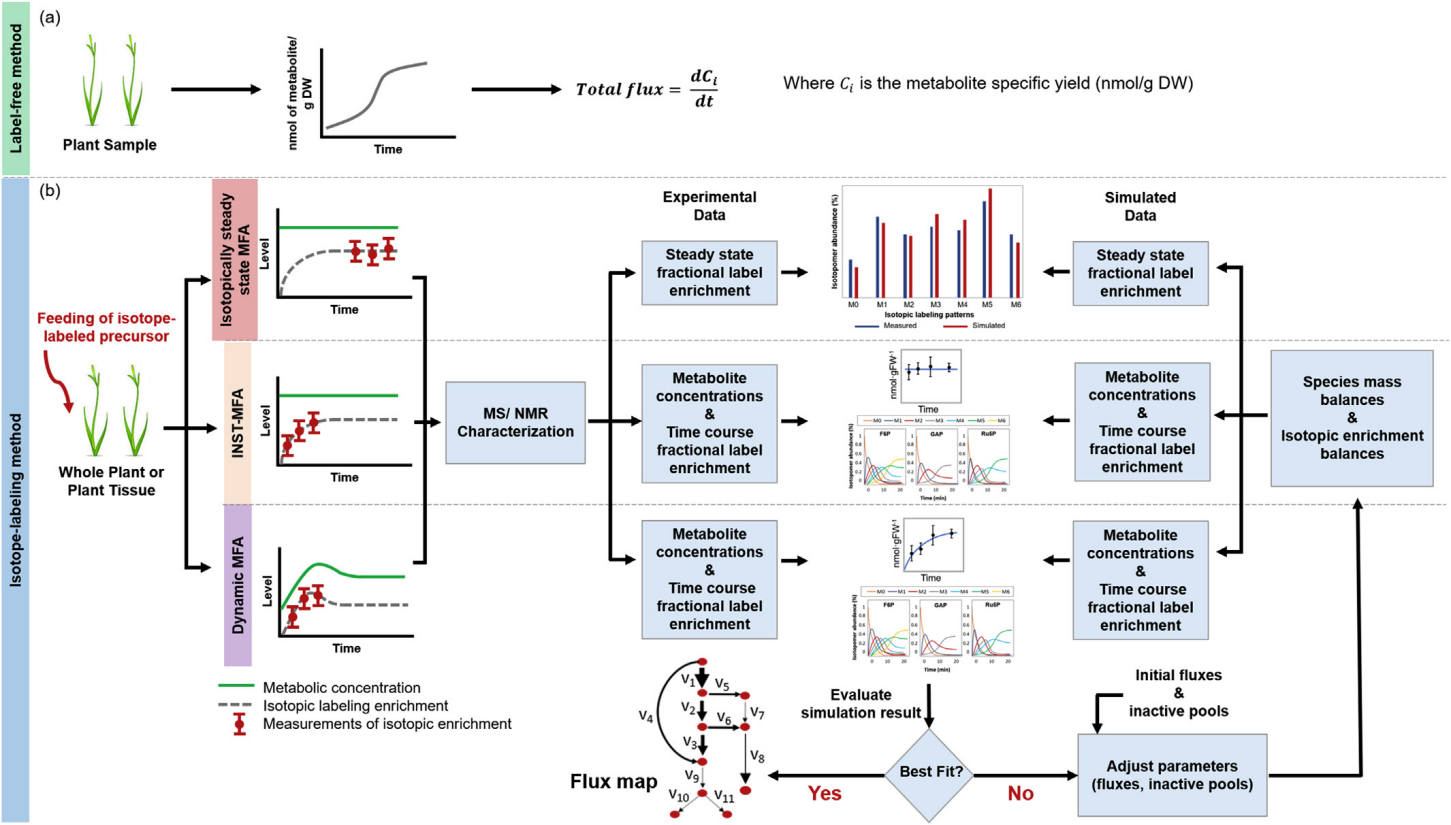
The overall framework of the (a) label-free and (b) isotope-labeling metabolic flux analysis (MFA) techniques. The isotope-labeling methods can be classified as isotopically steady state MFA, isotopically non-steady state MFA (INST-MFA), and dynamic MFA.

Intuitively, analyzing the flux in secondary metabolic pathways with little branching seems straightforward. If the pathways are not cyclic, and assuming the turnover of the final products is small relative to their synthesis, then the flux can be calculated by following the concentrations of all the end products with time (Fig. 3(a)). Unlike primary metabolites, the carbon backbones of the secondary metabolites are seldom rearranged. Hence, in spite of the high diversity of secondary metabolites, they can be grouped into families by structural similarity. By summing up the individual metabolic fluxes that share a common precursor, the upstream flux value can be readily calculated (Morgan and Shanks, 2002). However, this approach has an inability to resolve fluxes between pathways that operate in parallel and converge downstream. For example, as shown in Fig. 1, the flux to the formation of methyl eugenol can be easily calculated by following its accumulation with time. However, it is impossible to gain the information about the contribution of the flux from the route of caffeoyl-CoA to feruloyl-CoA and the flux via ferulic acid to feruloyl-CoA. Therefore, other techniques must be implemented to handle this complexity of the metabolic network.

### 2.2 Metabolic flux analysis with stable isotope labeling

There are a substantial number of papers that feed isotopically labeled precursors such as ^13^C-labeled or ^2^H-labeled substrates to plants and profile the changes to metabolite concentrations and isotopic incorporation. However, few of these studies actually use this data to calculate fluxes. The flux calculations will depend on whether steady-state labeling is achieved and whether the pool sizes remain constant (Fig. 3(b)). The experimental data needed are the intracellular metabolite concentrations, and the levels of isotope enrichment with nuclear magnetic resonance (NMR) or mass spectrometry (MS) which is typically coupled with an upstream separation technique. The experimental details and analysis of the labeling patterns in plants have been reviewed elsewhere (Ratcliffe and Shachar-Hill, 2006).

#### 2.2.1 Steady state MFA

Steady-state isotopic labeling based metabolic flux analysis (^13^C MFA) measures the isotopic abundance of metabolic intermediates or end-products at metabolic and isotopic steady-state to obtain flux maps by the workflow shown in Fig. 3(b). The steady-states referred to here are when the metabolite level and the isotopic abundance of individual carbons do not statistically change with time. The reactions through which the isotopically labeled substrates proceed will determine the distribution of label in the end products. This approach is capable of producing flux maps for pathways containing metabolic cycles as well as reverse and exchange fluxes. In addition, it is possible to determine the flux splits between two parallel pathways at metabolic branch points provided that there is an asymmetry between the two pathways leading to different labeling patterns of the end products (Stephanopoulos, 1999).

It is worth mentioning that when applying steady-state MFA, the time period required to achieve both steady-states has to be short to avoid significant metabolic shifts (Schwender, 2008). However, the typical experimental periods required for plant metabolism to reach isotopic steady state after labeled precursor feeding can be long, rendering it difficult to routinely apply. This is also the reason why, if ^13^C MFA is applied to plants, the general focus has been around central carbon metabolism (Kruger et al., 2012) in which the steady state can be reached within hours (Masakapalli et al., 2010). Additionally, a challenge for applying MFA utilizing isotopically labeled external substrate to plants in autotrophic metabolism is the assimilation of natural abundance CO_2_ which would dilute the fed labeled carbon in the end products. Moreover, labeling with CO_2_ to isotopic steady state would eventually result in uniform and uninformative labeling for autotrophic cells since they assimilate carbon solely from CO_2_ (Shastri and Morgan, 2007). Therefore, conventional steady state ^13^C MFA is not capable of quantifying pure autotrophic metabolic flux. As a result, previous steady-state ^13^C MFA studies of plants have been generally limited to cases where substrates such as isotopically labeled glucose is fed to plant tissues under mixo- or heterotrophic conditions. For example, the discovery of two alternative pathways for IPP biosynthesis exist was shown by feeding [1–^13^C] glucose to plants grown under heterotrophic conditions. They observed different ^13^C labeling patterns in cytosol derived sterols compared to plastid derived isoprenoids indicating the involvement of separate MVA and MEP pathways (Lichtenthaler et al., 1997). However, ^13^C MFA cannot resolve the metabolic pathways in which no rearrangement of the labeled core carbon structure occurs, which is often the case in secondary metabolism (Fig. 1). To address and overcome these concerns, isotopically nonstationary or dynamic MFA is used.

#### 2.2.2 Isotopically nonstationary MFA (INST-MFA)

Isotopically nonstationary MFA (INST-MFA) is a method that has successfully been employed for analyzing central carbon metabolism in microorganisms (Cheah and Young, 2018; Wiechert and Nöh, 2013). For organisms growing under photoautotrophic conditions, a step change is made in the feed from natural abundance CO_2_ to enriched ^13^C–CO_2_. Next, biological samples are quenched and intracellular metabolites are extracted over a time course. Then transient labeling patterns of intermediates are analyzed to estimate metabolic fluxes (Fig. 3(b)). In this approach, the metabolic fluxes and metabolite pool sizes are not perturbed by the introduction of ^13^C tracer and remain constant. In the work by Young and coworkers (Young et al., 2011), they showed the combination of INST-MFA with GC-MS and LC-MS/MS for analyzing the dynamic isotope labeling trajectories of central metabolic intermediates can precisely quantify the photoautotrophic fluxes in cyanobacteria. This approach was extended to quantitatively describe fluxes in central carbon metabolism and photosynthesis in *Arabidopsis thaliana* rosettes under different light conditions by administering ^13^CO_2_ to whole plants (Ma et al., 2014).

#### 2.2.3 Dynamic MFA

Similar to INST-MFA, dynamic MFA measures transient isotope abundances of metabolites following addition of an exogenously fed isotopically labeled precursor. In this type of experiment, time course measurements of pool sizes of metabolic intermediates are also performed because of the perturbations resulting from the external feed. To detect significant labeling in downstream metabolites, it is often necessary to feed levels of substrate that affect intracellular pool sizes. This requires the dynamic measurement of both intermediate pool sizes and the respective isotopic label (Fig. 3(b)). This type of experiment has been used to analyze fluxes in secondary metabolic networks (Boatright et al., 2004; Cin et al., 2011; Matsuda et al., 2005, 2003; Okazaki et al., 2004; Orlova et al., 2006; Tieman et al., 2006; Zhang et al., 2015). As a specific example, Boatright et al. (2004) fed ring-labeled phenylalanine (^2^H_5_-Phe) to Petunia flowers and measured the time course of label, internal pools, and emitted volatiles from the benzenoid network (Fig. 1). From this data, they calculated metabolic flux and found that both the β-oxidative and non-β-oxidative pathway contribute to the biosynthesis of benzoic acid (BA). The following work (Orlova et al., 2006) that generated a transgenic plant in which the expression of benzoyl-CoA:benzyl alcohol/phenylethanol benzoyltransferase (BPBT) was reduced or eliminated confirmed the role of benzylbenzoate as an intermediate in the BA synthesis network. By comparing the flux maps generated with the control and the transgenic plants under the light and dark conditions, the greater contribution of benzylbenzoate to BA synthesis was observed in the light. Further, the suppressed flux to benzylbenzoate resulted in a decrease of the total flux to BA and an increase of the flux through the non-β-oxidative pathway.

#### 2.2.4 Metabolic channeling and compartmented metabolite pools

The analysis of fluxes by ^13^C-MFA can provide evidence for the existence of metabolite channeling and/or inactive metabolite pools. Metabolite channeling is a process by which intermediates are transferred directly between catalytic sites in multi-enzyme complexes without diffusion into the bulk phase of the cell. Hence, intermediates outside of the channel are modeled as metabolically inactive pools and remain unlabeled, causing the phenomenon of the label abundance of the upstream precursors to be lower than that of the downstream products. Similarly, the existence of an inactive pool in another location can result in the same observation. Because of the subcellular compartmentation in plant cells, there may exist a separate metabolite pool outside the compartment where the reaction is taking place. The intermediates in the separate metabolite pool are not able to incorporate the isotope from the labeled precursor in the time scale of the labeling experiment. However, the metabolites extracted from the plant cells will mix the active and inactive metabolite pools, consequently diluting the ^13^C-labeling level. By including dilution parameters in the isotopomer reaction models, the contributions of metabolic channeling (Young et al., 2011) and inactive pools (Ma et al., 2014) were estimated. We should note that steady-state MFA has also been used to detect metabolic channeling in plant cultures (Williams et al., 2011).

## 3 Future directions for MFA in plants

There is much still to be discovered about the function, localization (Stavrinides et al., 2015) and regulation of enzymes in metabolic pathways that will result in modifying our understanding of the connections between secondary metabolites (Barros et al., 2019; Wurtzel and Kutchan, 2016). A recent example is the discovery of a cytosolic pathway for phenylalanine biosynthesis that runs parallel to the plastidic pathway (Qian et al., 2019). Therefore, implementing pathway discovery tools is critical to describe the correct metabolic network structure.

In addition, there would be a substantial benefit to quantify fluxes in primary metabolism which provide precursors for secondary metabolism. As stated earlier, this will have to be performed with separate MFA experiments and modeling (Sriram et al., 2007). This knowledge will aid in understanding how environmental conditions and developmental stages affect primary metabolism, and consequently the synthesis of the precursors of secondary metabolic pathways. Combining flux maps with other systems biology measurements (Rai et al., 2017), mechanistic models of flux control such as kinetic models (Guo et al., 2018) can be formulated that have both explanatory and predictive powers.

## 4 Summary

This review described the adaptation of tools that have been developed and applied previously to microbial or mammalian cell cultures. Improvements in precise quantification of pool sizes in specific tissues, cell types, and even subcellular localization will enable the improved estimation of fluxes (Delfin et al., 2019). We envision the quantification of metabolic fluxes will have application in the burgeoning field of plant synthetic biology (Fesenko and Edwards, 2014; Fuentes et al., 2016; Küken and Nikoloski, 2019; Liu and Stewart, 2015) and enable the rational modification of metabolic networks in plants, resulting in the increase in yield of secondary metabolites as well as tailoring the product selectivity. Both basic science and applied studies of plant secondary metabolism should greatly benefit from MFA under a variety of relevant environmental conditions.

## Declaration of competing interest

The authors declare that they have no known competing financial interests or personal relationships that could have appeared to influence the work reported in this paper.
